# The combination of microbial agent, organic fertilizer, and silica powder enhances oat yield and quality through regulating rhizosphere processes

**DOI:** 10.3389/fmicb.2026.1901736

**Published:** 2026-10-15

**Authors:** Ping Chen, Yuanyuan Luo, Lingfang Ma, Mengmeng He, Hongxiu Ma, Wenli Ma, Quan Sun

**Affiliations:** 1 College of Forestry and Prataculture, Ningxia University, Yinchuan, Ningxia, China; 2 Ningxia Reclamation, Agricultural, Forestry, and Animal Husbandry Technology Promotion and Service Center, Yinchuan, Ningxia, China; 3 Key Laboratory of Grassland Ecological Protection and Native Grass Germplasm Innovation of Shanxi, Shanxi Agricultural University, Taigu, China

**Keywords:** biosilica fertilizer, microbial community, oats, salinized soil, soil enzyme activity

## Abstract

**Aims:**

Soil salinization is a global challenge for sustainable agricultural development. Composite modifiers have the potential to boost the agricultural productivity of salinized soils. However, it’s still unclear whether it outperforms single modifiers in regulating rhizosphere soil properties, microbial communities, and forage yield and quality of different oat varieties (*Avena sativa* L.) and the mechanisms.

**Methods:**

In this field experiment in 2023–2024 in the arid western China, five modifier treatments, namely CK (control group without any modifier), TJ (microbial agent), TF (organic fertilizer), TG (silica powder), and TJFG (microbial agent + organic fertilizer + silica powder), were designed for two oat varieties (TY 60, Tianyan 60; MST 2, Musite 2). Then, their effects on salinized soil properties, rhizosphere microbial communities, and the interaction between soil nutrient cycling and microbial composition were explored.

**Results:**

The crude protein (CP) content of the two oat varieties peaked under the TJFG treatment. The rhizosphere soil NH_4_^+^-N, available phosphorus (AP), available potassium (AK), organic matter (SOM), and water content (SWC) of the TJFG treatment were significantly higher than those of CK, while the soil pH and NO_3_^−^-N content were significantly lower. In addition, the AP content of the TJFG treatment was 70.17, 44.91, and 66.93% (average over 2 years) higher than that of the TJ, TF, and TG treatments, respectively.

**Conclusion:**

The application of TJFG improved the diversity of bacterial and fungal communities as well as the activities of phosphatase (ATP), urease (UE), and catalase (CAT), thereby promoting soil nutrient transformations and enhancing soil fertility, oat yield, and quality. Chloroflexota and Ascomycota were the key microbial phyla to regulate oat yield. Organic matter, AK, AP, and CAT activity were the main soil factors affecting yield. Overall, MST 2 has greater growth potential and more value for promotion and application than TY 60. This study provides an important reference for salinized land use, ecological health maintenance, and sustainable agricultural production in arid regions.

## 1 Introduction

Soil degradation caused by climate change, agricultural production, and intensive land use has become increasingly serious in recent years (Karlen and Rice, 2015). Soil salinization is one of the key factors leading to soil degradation. It significantly restricts agricultural productivity and ecosystem sustainability (Sofy et al., 2020). It is estimated that the area of salinized lands in the world has reached about 1.1 billion hectares. Influenced by topography, climate change, and human activities, soil salinization shows a continuously increasing trend, posing a serious threat to global agricultural production and ecosystem health (Zhang et al., 2020a). Currently, salinized lands reach approximately 99.13 million hectares worldwide, accounting for about 10% of the total land area (Liu et al., 2023). Due to China’s large population and limited arable land, salinized land has become a potential arable land resource. Various remediation measures, including biological and chemical measures, have been implemented on salinized lands. Although these measures can improve the soil environment, they still have certain limitations, such as secondary pollution, and their long-term effects need further verification. In recent years, an increasing number of studies have introduced high-value-added salt-tolerant crops to enhance the productivity of salinized lands (Zhao et al., 2020b; Yu et al., 2023), providing a new path for the utilization of this land resource. Selecting salt-tolerant crop varieties and matching them with the right farming practices have become really important for sustainably using salinized soil.

Oat (*Avena sativa* L.) is a cereal crop that originated along the Mediterranean coast and in the Middle East. It is a globally important healthy food and forage crop due to its rich nutrition, high-quality feed value, and strong environmental adaptability. In recent years, oats have been widely planted in the salinized lands in arid northwest China (Sánchez-Martín et al., 2020; López-Bellido and López-Bellido, 2015). However, the suitable habitats for different oat varieties vary. Therefore, it is necessary to match different oat varieties with the specific conditions of different regions. Furthermore, salinized lands usually have poor soil structure and low fertility, restricting crop growth and yield formation (Bai et al., 2025). Against this background, developing environmentally friendly soil modifiers to alleviate salt stress, enhance soil fertility, and promote oat yield formation is increasingly necessary. It is an important way to relieve the contradiction between the reduction in arable lands and increasing food demands (Kopittke et al., 2019). Because of the synergistic effects of composite modifiers, they are considered promising (Li et al., 2026; Wang et al., 2022). However, it’s still unclear how different oat varieties respond to composite modifiers, and whether the components of composite modifiers have a synergistic effect on rhizosphere processes.

The combined application of organic and inorganic fertilizers is an effective way to improve salinized soils (Sun et al., 2022). Organic modifiers improve soil structure and rapidly supply fertilizer nutrients (Li et al., 2021b). Silica powder, as an inorganic soil modifier, adjusts soil pH, promotes the formation of aggregated soil structure, and enhances the bioavailability of nutrients such as phosphorus and potassium (Neu et al., 2017; Wang et al., 2021). In addition, silicon can be deposited in plants to form a silicide layer, enhancing the mechanical strength of stems and resistance to abiotic stresses such as drought and high temperatures (Cooke and Leishman, 2016; Katz et al., 2021). Previous studies have shown that compared with organic fertilizer, the combination of organic fertilizer and silica powder can significantly improve soil structure, pH, and microbial function (Zhao et al., 2020a), increasing soil quality and forage yields (Sun et al., 2022). Microbial fertilizers are environmentally friendly, produced through microbial fermentation (Bagga et al., 2024). Rich functional microorganisms can promote soil nutrient cycling and crop nutrient absorption, enhancing soil quality and productivity (Kumar et al., 2021). A field experiment has shown that the application of microbial agents alleviates salt stress by reducing soil salinity and pH (She et al., 2018), thus increasing crop yields. Notably, with the input of exogenous nutrients and microorganisms, the diversity and structure of soil bacterial and fungal communities change significantly (Jinu and Kee-Choon, 2016). Particularly, the abundance of bacteria involved in nutrient cycling and organic matter degradation increases significantly (Fan et al., 2019). It can be seen that different types of modifiers have various effects on soil microbial community structure (Li et al., 2021a). However, current research focuses on the organic–inorganic fertilizer combined application and microbial agents. There is still a lack of research on the interaction mechanism among the three.

Soil enzymes regulate soil nutrient cycling and microbial community function, directly reflecting the resource limitations faced by microbial metabolism (Zheng et al., 2023; Liu et al., 2024). Studies have shown that the application of organic fertilizer can affect soil microbial community structure and alleviate resource limitations. This is mainly because organic fertilizers provide easy-decomposing carbons and multiple nutrients, significantly changing the resource acquisition strategies and extracellular enzyme secretion of microorganisms (Luo et al., 2023; Yang et al., 2024). Changes in enzyme activity are often closely related to the adjustment of microbial metabolic strategies and may promote the decomposition of organic matter and nutrient transformation (Chen et al., 2023). However, the specific mechanisms of organic–inorganic fertilizers alleviating resource constraints and regulating soil microbial communities are still unclear, particularly under salt stress conditions.

In summary, existing studies focus on the effects of single or organic–inorganic combined modifiers. There is still a lack of systematic analysis on how a composite modifier made of microbial agent, organic fertilizer, and silica powder jointly regulates the rhizosphere microecology of different oat varieties. This limits the precise application of salinized soil remediation techniques. The purpose of this study is to elucidate the effects of different soil modifiers on the relationships of rhizosphere soil microbial communities, nutrient availability, and enzyme activities with the yield and quality of different oat varieties. This study hypothesized that (1) There might be an optimal soil modifier that can improve the microbial community structure of oat rhizosphere soil in arid regions; (2) The combination of microbial agent, organic fertilizer, and silica powder might increase soil enzyme activity through improving soil fertility compared with single modifiers; (3) The application of composite modifier might significantly enhance the soil quality of salinized soil and crop yields compared with single modifiers.

## 2 Materials and methods

### 2.1 Experimental site

The experimental site is located in Qianjin Farm, Shizuishan City, Ningxia Hui Autonomous Region, China (106° 43′ E, 38° 82′ N), with a temperate continental climate. The average annual temperature was 9.9 °C. The average annual precipitation was 188.8 mm (mainly concentrated in July and August). The average annual evaporation was 2,512.6 mm. Figure S1 shows the weather conditions during the oat growing seasons in the region. The soil used in the experiment was moderately saline (Ministry of Agriculture and Rural Affairs of the People’s Republic of China, 2019). Key salinization indicators are shown in Supplementary Table S1. The organic matter content in the plow layer (0–20 cm) was 8.08 g/kg. The total nitrogen content (TN) was 0.47 g/kg, the available phosphorus (AP) content was 5.47 mg/kg, and the available potassium (AK) content was 252.6 mg/kg, indicating low soil fertility. The groundwater level was 1.5 m. Based on our previous study on oats’ salt stress tolerance during germination (Chen et al., 2025), MST 2 is a salt-tolerant genotype, while TY 60 is a salt-sensitive genotype. Both are widely grown in the salinized areas of Ningxia, China, indicating a high degree of representativeness. Therefore, this study chose these two varieties to analyze how different oat genotypes respond to the composite modifier.

This experiment started on March 1, 2023. A completely randomized block design was adopted. Five treatments were designed in this experiment, namely CK (control group without any soil modifier), TJ (microbial agent), TF (organic fertilizer), TG (silica powder), and TJFG (microbial agent + organic fertilizer + silica powder). All plots received the same amount of nitrogen, phosphorus, and potassium fertilizers, with N, P_2_O_5_, and K_2_O being 90, 177, and 60 kg/ha, respectively. Superphosphate (46% P_2_O_5_) and potassium sulfate (52% K_2_O) were evenly applied to the soil before sowing. Urea (46% N) was topdressed before the jointing stage. Each treatment had three repetitions. The area of each plot was 36 square meters (4.5 m × 8 m), and the plot spacing was 1 m. The sowing rate was 225 kg/ha, and the sowing depth was 3 cm. The row spacing was 30 cm. Microbial agent (Bacillus ≥200 million/mL, provided by Beijing Huimao Biotechnology Co., Ltd., China), organic fertilizer (organic matter ≥30% (calculated on a dried basis); N + P_2_O_5_ + K_2_O content ≥4.0%; mainly cow dung; provided by Ningxia Nongken Bio-Fertilizer Factory, China), silica powder (silicon dioxide ≥90%; provided by Ningxia Qiyuan Biotechnology Co., Ltd., China). The microbial agent was a water-based solution, and the application rate was 30 kg/ha. It was diluted with water at a ratio of 1:500 before being sprayed onto the soil surface. The organic fertilizer was granular, with an application rate of 12 tons per hectare. The application rate of silica powder was 105 kg per hectare. Organic fertilizer and silica powder were evenly spread on the soil surface and then mixed with the topsoil (0–15 cm layer). Other management practices were consistent with those in local fields.

### 2.2 Soil and plant sampling

At harvest (October 2023 and October 2024), five sampling points were selected in each plot, and one oat plant was selected at each point. After removing impurities such as plant residues and animal remains, rhizospheric soil was collected (Ren et al., 2022). Then, the five samples of each plot were mixed. Before analysis, all soil samples were passed through an 8 mm sieve to remove stones and plant debris. Then, part of the samples was air-dried, ground, and sieved to determine soil physicochemical properties and enzyme activity (Wei et al., 2020). The other part was transferred into a 10 mL sterile centrifuge tube and stored at −80 °C for microbial community analysis. At the same time, a small area (1 m × 1 m) was selected at each sampling point for mowing using a sickle, followed by weighing to obtain the fresh weight. The collected fresh samples were brought back to the laboratory and dried to a constant weight. Finally, the hay yield was obtained by weighing.

### 2.3 Determination of soil physicochemical properties and enzyme activity

#### 2.3.1 Soil physicochemical properties

Soil pH was determined using a pH meter (Sartorius PB-10, Germany) at a soil: water ratio of 1: 2.5 (He et al., 2023). Soil moisture content was determined by the weighing method (Zhang et al., 2020b). Soil organic matter (SOM) content was determined by potassium dichromate oxidation (Walkley and Black, 1934). Soil available phosphorus (AP) was extracted with sodium bicarbonate, and its content was determined by a spectrophotometer (UV-1800, Shimadzu, Japan; Olsen et al., 1954). Soil available potassium (AK) was extracted with ammonium acetate, and its content was determined using a flame photometer (M410, Sherwood Scientific Ltd., UK; Zhao et al., 2023). Soil ammonium nitrogen (NH_4_^+^-N) and nitrate nitrogen (NO_3_^−^-N) were extracted with 0.5 M K_2_SO_4_ (1: 5, w/v), and their contents were determined by colorimetry (Mahmood et al., 2016).

#### 2.3.2 Soil enzyme activity

Soil alkaline phosphatase (ALP) activity was determined using disodium phenyl phosphate-based colorimetry, with ALP activity expressed as milligrams of phenol hydrolyzed in 1 g of air-dried soil within 24 h. By using a borate buffer (pH 10.0) and disodium phenyl phosphate as the substrate, incubation was carried out at 37 °C for 12 h. Soil urease (UE) activity was determined using sodium phenolate–sodium hypochlorite-based colorimetry. The UE activity was expressed as milligrams of ammonia produced in 5 g of soil within 24 h. Using a citrate buffer (pH 6.7) and a 10% urea solution as the substrate, incubation was carried out at 37 °C for 24 h. Soil catalase (CAT) activity was determined using a 0.1 mol L^−1^ KMnO_4_ titration method, with CAT activity expressed as milliliters of KMnO_4_ consumed in 5 g of soil within 24 h. This measurement used a phosphate buffer (pH 7.8), with hydrogen peroxide as the substrate, followed by back-titration with KMnO_4_. Enzyme activities were quantified by plotting standard curves. The units of ALP, UE, and CAT activities were U g^−1^ dry soil (Sun et al., 2022).

### 2.4 Determination of quality

After determining the hay yield, the dried plant samples were crushed and passed through a 1 mm sieve to determine quality indicators. The crude protein (CP) content was determined by the Kjeldahl method (Wang et al., 2023). The contents of neutral detergent fiber (NDF) and acidic detergent fiber (ADF) were determined by the methods of Van Soest et al. (1991). The relative feeding value (RFV) was calculated using the following formula (Ferreira et al., 2015):

RFV=120/NDF×(88.9–0.779×ADF)/1.29

### 2.5 DNA extraction, shotgun metagenomic sequencing, and bioinformatic analysis

Total genomic DNA of rhizosphere soil microorganisms was extracted using the QIAamp soil DNA extraction kit (QIAGEN, Hilden, Germany). The concentration and integrity of DNA were analyzed using a NanoDrop 2000 spectrophotometer (Thermo Fisher Scientific, Wilmington, DE, USA) and 1% agarose gel electrophoresis. Qualified DNA was randomly fragmented using a Covaris S220 ultrasonicator (Covaris, Woburn, MA, USA), purified with magnetic beads, and then used to construct paired-end sequencing libraries with the Illumina TruSeq Nano kit (Illumina, San Diego, CA, USA). Sequencing was performed on the Illumina NovaSeq 6000 platform (Illumina, San Diego, CA, USA; 150 bp paired-end reads), and each sample reached sufficient sequencing depth. Raw FastQ sequences were quality-filtered with Trimmomatic v0.36 to remove adapters and low-quality reads. Bowtie2 v2.2.9 was used to remove contaminant reads by aligning to the host plant genome, yielding clean valid sequences. MEGAHIT v1.1.2 was used to assemble these valid sequences, and core assembly metrics such as total scaffold count and scaffold N50 were analyzed to evaluate assembly quality. Open reading frames (ORFs) were predicted from assembled sequences using Prodigal v2.6.3. Non-redundant gene sets were constructed with CD-HIT v4.5.7 based on the thresholds of 95% sequence similarity and 90% coverage for shorter sequences, and clean reads from each sample were mapped to the gene set using Bowtie 2 to calculate gene abundance. This gene set analysis was used solely for downstream functional gene annotation and not for analyzing microbial community structure.

In this study, two species-annotation strategies were used for cross-validation: MetaPhlAn4 v4.0 served as the primary tool to directly quantify species from sequencing reads, supporting phylum-level species abundance statistics, α-diversity calculations, and non-metric multidimensional scaling (NMDS) based on Bray–Curtis similarity. Species annotation, which involved aligning the non-redundant gene set to the NR database, was used only as a supplement for functional analysis. Using the standardized species abundance matrix from MetaPhlAn 4, Chao1, Shannon, Simpson, and ACE indices were calculated. The raw sequencing data were uploaded to the NGDC Genome Sequence Archive (GSA) database under the accession number: CRA033617. R packages (version 4.3.1) were used to perform principal component analysis (PCA) on species and functional abundance and to compute Bray-Curtis distance matrices for principal coordinate analysis (PCoA) and NMDS. They were also used for data visualization and plotting.

### 2.6 Data analysis

All data were organized using Microsoft Excel 2016. Statistical analysis was conducted with SPSS 17.0. The experimental data were grouped by year and variety, and a one-way ANOVA was performed for each year–variety combination. When there was a significant difference at the *p* < 0.05 level, the least significant difference (LSD) test was used for multiple comparisons. An independent samples-based t-test was used to compare differences between oat varieties in the same year. Charts were drawn using OriginPro 2024. Dominant bacterial and fungal phyla (relative abundance >3%) were selected. Distance-based redundancy analysis (RDA) of soil environmental parameters and microbial community structure was completed in Canoco software 2020 (v5.12). The results of correlation analysis between soil microbial community structure and rhizosphere soil physicochemical properties and enzyme activities, as well as the Mantel test, were drawn using the “ggcor” package in R (version 4.3.1). The random forest model was constructed to predict the relative importance of soil microbial abundance, diversity, and physicochemical properties for oat yield. The analysis used the randomforest R package, and the significance of each predictor was evaluated using the rfpermute R package. Variable importance was assessed by percentage increases in mean squared error (MSE): Higher MSE% values indicated greater importance.

## 3 Results

### 3.1 Oat yield and quality

Modifiers significantly affected the oat yield, and the yield of variety MST 2 was significantly higher than that of TY 60 in 2024 (Figure 1). The yield of variety MST 2 of the TF and TJFG treatments was 21.51 and 25.23% (two-year average) higher than that of CK, respectively (*p* < 0.05). There was no difference in oat yield between the TJFG and other modifier treatments (*p* > 0.05).

**Figure 1:**
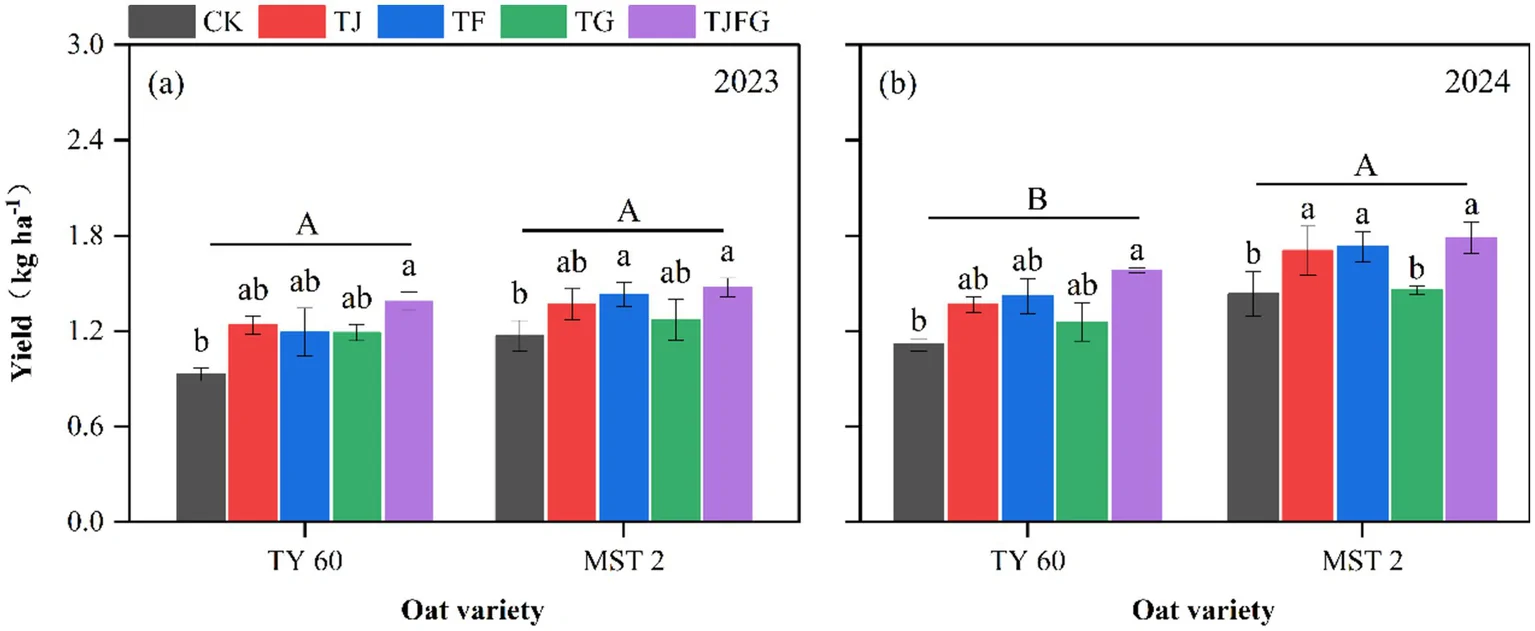
Effects of different modifiers on oat yield in 2023 and 2024. TY 60, Tianyan 60; MST 2, Musite 2; CK, control without any modifier; TJ, microbial agent; TF, organic fertilizer; TG, silica powder; TJFG, microbial agent + organic fertilizer + silica powder. Different uppercase letters indicate significant differences between oat varieties (*p* < 0.05); different lowercase letters indicate significant differences between modifier treatments (*p* < 0.05). Bars represent the standard errors (*n* = 3). The same below.

In 2024, variety significantly affected the contents of RFV, NDF, and ADF, while there was no difference in the CP content among varieties (*p* > 0.05; Figure 2). The CP content of varieties TY 60 and MST 2 of the TF, TG, and TJFG treatment was 12.78–27.44% and 22.02–35.08% (two-year average, the same below) higher than that of CK (*p* < 0.05). The maximum CP content of TY 60 (9.28%) and MST 2 (9.42%) was found in the TJFG treatment. The RFV value of both varieties responded differently to modifiers. The RFV value of variety TY 60 of the CK and TJFG treatments was higher than that of the TF and TG treatments, but there was no difference between the CK and TJFG treatments (*p* > 0.05). The variety MST 2 of the TJFG treatment had the maximum RFV, which was 0.47–26.49% higher than that of other treatments. The ADF content of both varieties of the TJFG treatment was significantly lower than that of other treatments.

**Figure 2:**
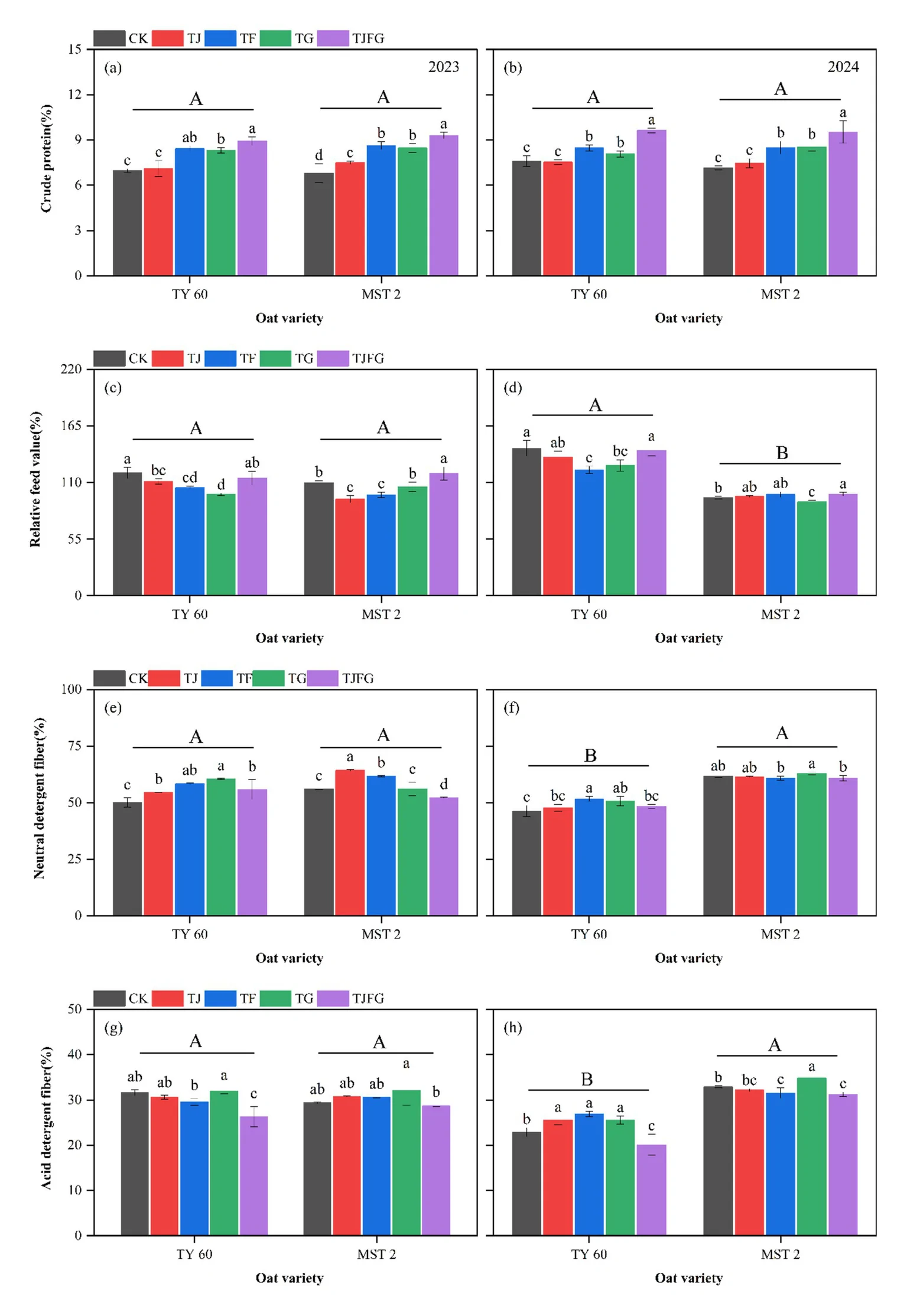
Effects of different modifiers on oat quality [crude protein content **(a,b)**, relative feeding value **(c,d)**, neutral detergent fiber **(e,f)**, and acidic detergent fiber **(g,h)**] in 2023 and 2024.

### 3.2 Physicochemical properties and enzyme activity of rhizosphere soil

Varieties significantly affected soil ammonium nitrogen (NH_4_^+^-N), nitrate nitrogen (NO_3_^−^-N), available potassium (AK), organic matter (SOM), and water content (SWC). However, the type of modifier significantly affected soil pH, AP, and SWC (Supplementary Table S2). MST 2 had a higher accumulation of NH_4_^+^-N and a smaller leaching loss of NO_3_^−^-N compared with TY 60 (Table 1). For the two varieties, the pH of the rhizospheric soil of the TJ, TF, and TJFG treatments was significantly lower than that of CK; particularly, the soil pH of the TJFG treatment was 4.13–10.11% lower (*p* < 0.05). The soil NH_4_^+^-N content of the TJFG treatment was 22.04–26.11% higher than that of CK (*p* < 0.05). The soil NO_3_^−^-N content of the TG and TJFG treatments was 31.59–48.63% and 53.10–66.22% lower than that of CK, respectively (*p* < 0.05). The contents of soil AP, AK, SOM, and SWC of the TJFG treatment were 137.65–158.28%, 10.71–23.78%, 11.05–251.3%, and 16.27–50.58% higher than those of CK, respectively (*p* < 0.05).

**Table 1:** Effects of different modifiers on the physicochemical properties of oat rhizospheric soil.

Year	Variety	Treatment	pH	NH_4_^+^-N	NO_3_^−^-N	AP	AK	SOM	SWC
2023	Tianyan 60	CK	8.74 ± 0.45a	1.31 ± 0.09b	19.37 ± 1.11a	8.76 ± 0.31c	258.69 ± 8.39c	6.46 ± 0.28c	12.05 ± 0.41d
		TJ	8.37 ± 0.40b	1.44 ± 0.11ab	16.05 ± 0.85bc	10.01 ± 0.49bc	263.78 ± 11.50bc	7.15 ± 0.41b	16.34 ± 0.98c
		TF	8.34 ± 0.24b	1.40 ± 0.21b	16.93 ± 0.96ab	11.58 ± 0.55b	273.67 ± 7.85ab	7.78 ± 0.33a	16.79 ± 0.63bc
		TG	8.67 ± 0.36a	0.94 ± 0.05c	13.21 ± 0.58c	11.37 ± 0.59b	257.17 ± 7.92c	7.13 ± 0.18b	17.14 ± 1.33b
		TJFG	8.38 ± 0.28b	1.64 ± 0.08a	6.06 ± 0.35d	19.65 ± 1.15a	279.44 ± 8.60a	7.88 ± 0.39a	17.81 ± 0.80a
	Musite 2	CK	8.77 ± 0.51a	6.77 ± 0.15d	4.67 ± 0.35a	8.45 ± 0.22d	216.45 ± 17.30c	8.27 ± 0.27c	16.52 ± 0.92e
		TJ	8.15 ± 0.43c	7.82 ± 0.33b	4.16 ± 0.71a	13.57 ± 1.14c	227.58 ± 10.53bc	8.84 ± 0.43ab	18.92 ± 1.65c
		TF	8.30 ± 0.47c	8.24 ± 0.30a	2.78 ± 0.10b	16.27 ± 0.64b	248.91 ± 11.51ab	9.03 ± 0.38a	17.94 ± 1.31d
		TG	8.53 ± 0.37b	7.36 ± 0.42c	2.28 ± 0.26b	12.13 ± 0.92c	228.70 ± 10.60bc	8.41 ± 0.34bc	18.99 ± 0.84b
		TJFG	7.99 ± 0.40d	8.36 ± 0.46a	2.19 ± 0.09b	19.53 ± 1.26a	259.05 ± 13.95a	9.27 ± 0.41a	19.54 ± 1.36a
2024	Tianyan 60	CK	8.55 ± 0.61a	1.42 ± 0.05b	16.97 ± 1.15a	9.29 ± 0.44b	297.98 ± 9.55b	7.17 ± 0.52c	12.95 ± 1.16d
		TJ	8.20 ± 0.32bc	1.57 ± 0.08ab	16.12 ± 1.44a	10.35 ± 0.52b	315.42 ± 15.27ab	8.14 ± 0.61b	17.61 ± 1.45bc
		TF	8.10 ± 0.36c	1.64 ± 0.05ab	12.76 ± 1.21b	13.45 ± 0.59b	310.14 ± 16.25ab	8.35 ± 0.35b	16.61 ± 2.11c
		TG	8.45 ± 0.52ab	1.53 ± 0.16ab	11.65 ± 0.65b	11.08 ± 0.33b	300.57 ± 10.38b	7.87 ± 0.28b	18.78 ± 1.05ab
		TJFG	8.20 ± 0.41bc	1.81 ± 0.11a	6.15 ± 0.35c	27.17 ± 1.78a	337.91 ± 11.85a	9.18 ± 0.32a	19.86 ± 2.01a
	Musite 2	CK	8.66 ± 0.44a	7.14 ± 0.35c	4.75 ± 0.31a	9.11 ± 0.52d	223.48 ± 10.39b	9.57 ± 0.67c	17.76 ± 0.93b
		TJ	8.19 ± 0.51b	7.66 ± 0.22bc	4.39 ± 0.56a	9.73 ± 0.36d	261.88 ± 12.82a	9.75 ± 0.41bc	18.48 ± 1.12ab
		TF	8.24 ± 0.33b	8.25 ± 0.42ab	2.86 ± 0.15b	18.25 ± 1.15b	273.34 ± 10.21a	10.15 ± 0.47ab	18.91 ± 0.95ab
		TG	8.29 ± 0.35b	7.32 ± 0.25c	2.56 ± 0.12bc	15.84 ± 1.11c	228.51 ± 9.52b	9.54 ± 0.32c	20.09 ± 1.34a
		TJFG	7.68 ± 0.41c	8.61 ± 0.41a	2.23 ± 0.22c	22.25 ± 1.58a	285.79 ± 13.43a	10.53 ± 0.65a	20.29 ± 1.63a

TY 60, Tianyan 60; MST 2, Musite 2; CK, control without any modifier; TJ, microbial agent; TF, organic fertilizer; TG, silica powder; TJFG, microbial agent + organic fertilizer + silica powder. NH_4_^+^-N, soil ammonium nitrogen; NO_3_^−^-N, soil nitrate nitrogen; AP, soil available phosphorus; AK, soil available potassium; SOM, soil organic matter; SWC, soil water content. Different lowercase letters indicate significant differences between modifier treatments (*p* < 0.05). The same below.

The rhizospheric soil CAT activity of MST 2 was significantly higher than that of TY 60 in all treatments; there were no differences in ALP and UE activity (Figure 3). The rhizospheric soil ALP and UE activities of the two varieties of the TJFG treatment were 26.05–44.70% and 23.46–25.87% higher than those of CK, respectively (*p* < 0.05). The CAT activity of the modifier treatments was 29.97–64.47% higher than that of CK (*p* < 0.05). Among them, the soil CAT activity of the TJ and TJFG treatments was higher than that of other treatments (*p* < 0.05), but there was no difference between the TJ and TJFG treatments (*p* > 0.05).

**Figure 3:**
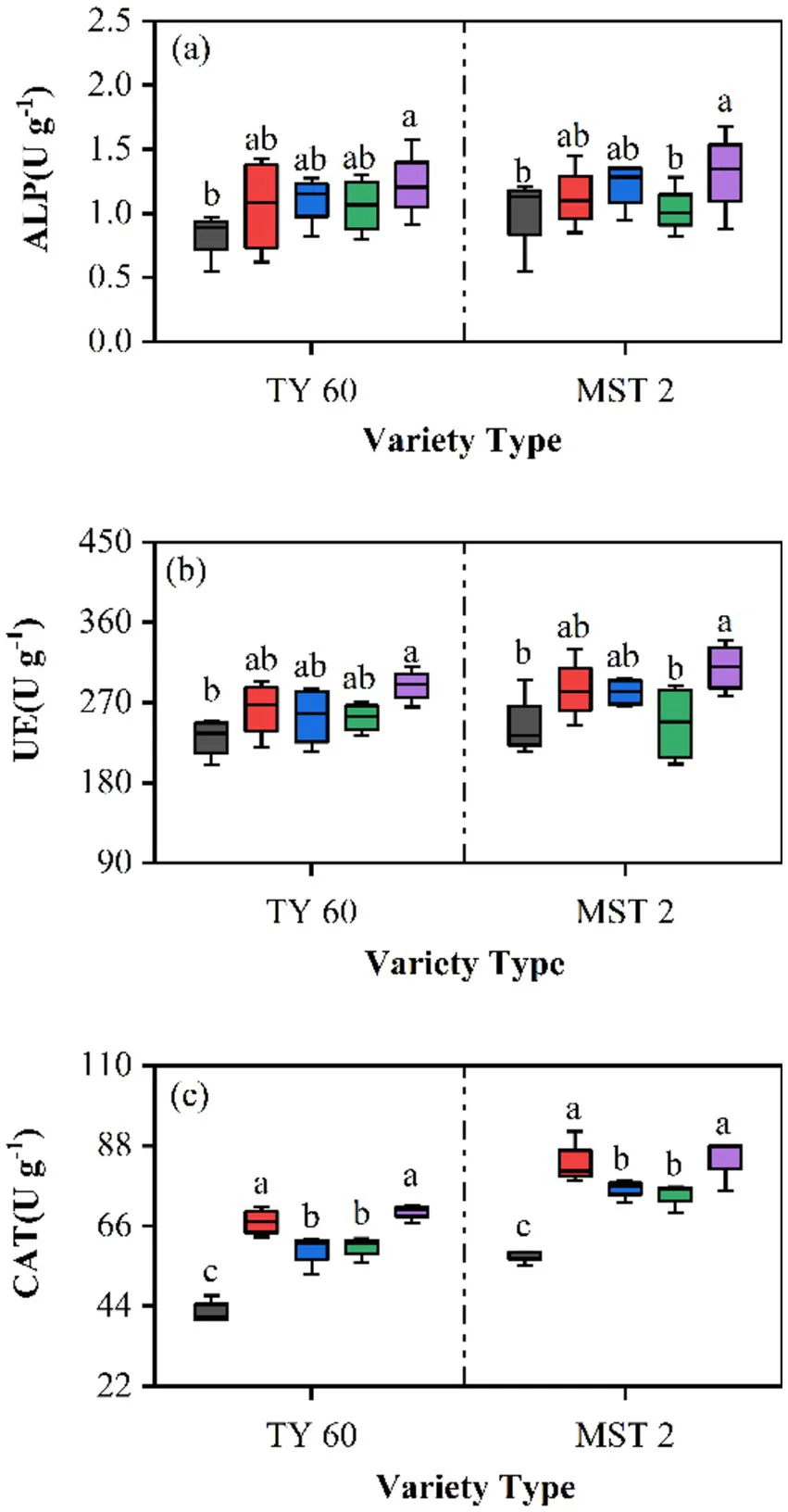
Effects of different modifiers on the rhizospheric soil enzyme activity. **(a)** Alkaline phosphatase (ALP); **(b)** Urease (UE); **(c)** Catalase (CAT).

### 3.3 Microbial community richness and diversity

The variety-modifier interaction significantly affected bacterial Chao1 and ACE indices and all fungal α diversity indices (Supplementary Tables S3, S4). The Chao1 index of the soil bacterial community of the TJFG treatment was 1.68–3.54% higher than that of CK (*p* < 0.05). The fungal Chao1 and ACE indices of the modifier treatments were 13.67–35.31% and 11.32–44.88% higher than those of CK (*p* < 0.05), respectively. Among them, the fungal Chao1 and ACE indices of the TJFG treatment were 22.74–35.31% and 9.78–10.99% higher than those of CK (*p* < 0.05), respectively.

### 3.4 Microbial community composition

The dominant bacterial phyla in oat rhizospheric soils in all treatments were Pseudomonadota (44.69–47.31%), Bacteroidota (13.03–15.94%), Actinomycetota (10.11–11.63%), Acidobacteriota (6.71–8.49%), and Gemmatimonadota (3.47–5.66%). These dominant bacteria accounted for more than 82% of the total bacterial sequences/reads. The relative abundances of these dominant bacteria in the rhizospheric soils of the modifier treatments were higher than those of CK (*p* < 0.05). In particular, the abundance of Bacteroidota peaked in the TJFG treatment, which was 111.92% higher than that of CK (Figure 4a).

**Figure 4:**
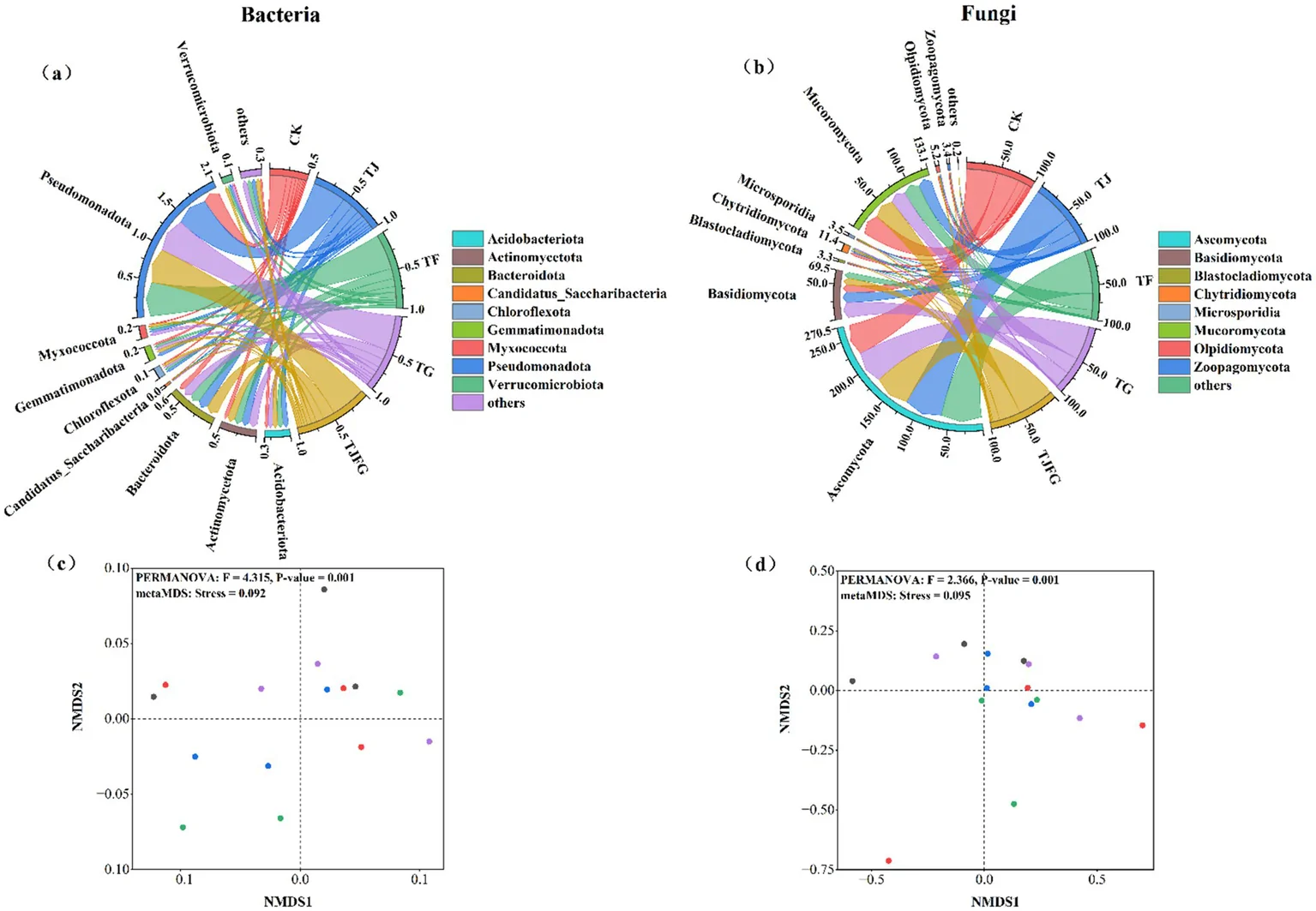
The relative abundance of the top 9 bacterial phyla **(a)** and the top 8 fungal phyla **(b)** in the rhizospheric soils of different oat varieties under different modifier treatments. Bray-Curtis NMDS (Nonmetric Multidimensional Scaling) analysis of changes in rhizosphere soil bacterial **(c)** and fungal **(d)** communities [based on OTU (Operational Taxonomic Unit) abundance and UniFrac distance].

The dominant fungi in oat rhizospheric soils were Ascomycota (46.40–62.21%), Mucoromycota (20.34–32.28%), and Basidiomycota (9.28–23.15%). The relative abundance of Ascomycota of the modifier treatments was 6.76–34.08% higher than that of CK (*p* < 0.05; Figure 4b). The relative abundance of Mucoromycota of the modifier treatments was 24.32–37.01% lower than that of CK (*p* < 0.05). Bray-Curtis NMDS analysis showed that soil bacterial and fungal community structures changed significantly with soil environmental changes (Figures 4c,d).

### 3.5 Relationship between soil microbial communities and soil physicochemical properties

Redundancy analysis (RDA) of the impact of soil physicochemical properties on microbial community structure (Figures 5a,b) showed that the first two axes, RDA1 and RDA2, explained 84.04 and 79.58% of the total variation in soil bacterial and fungal community structure, respectively (*p* < 0.05). The relative abundance of dominant bacterial phyla was positively correlated with soil enzyme activity and the contents of SWC, AP, AK, SOM, and NH_4_^+^-N, and negatively correlated with soil pH and NO_3_^−^-N content (Figure 5a). The fungal community also showed similar results (Figure 5b). The Mentel test results (Figure 5c) showed that soil bacterial community structure was positively correlated with the contents of NO_3_^−^-N, AK, and SOM (*p* < 0.05), soil pH (*p* < 0.01), SWC (*p* < 0.01), and CAT activity (*p* < 0.01). Soil fungal community structure was positively correlated with soil physicochemical properties except for AP content (*p* < 0.05); in particular, it was positively correlated with soil CAT activity (*p* < 0.01). The random forest analysis (Figure 5d) found that SOM content had the greatest impact on oat yield, followed by the relative abundance of Chloroflexota and Ascomycota, AP content, AK content, and CAT activity.

**Figure 5:**
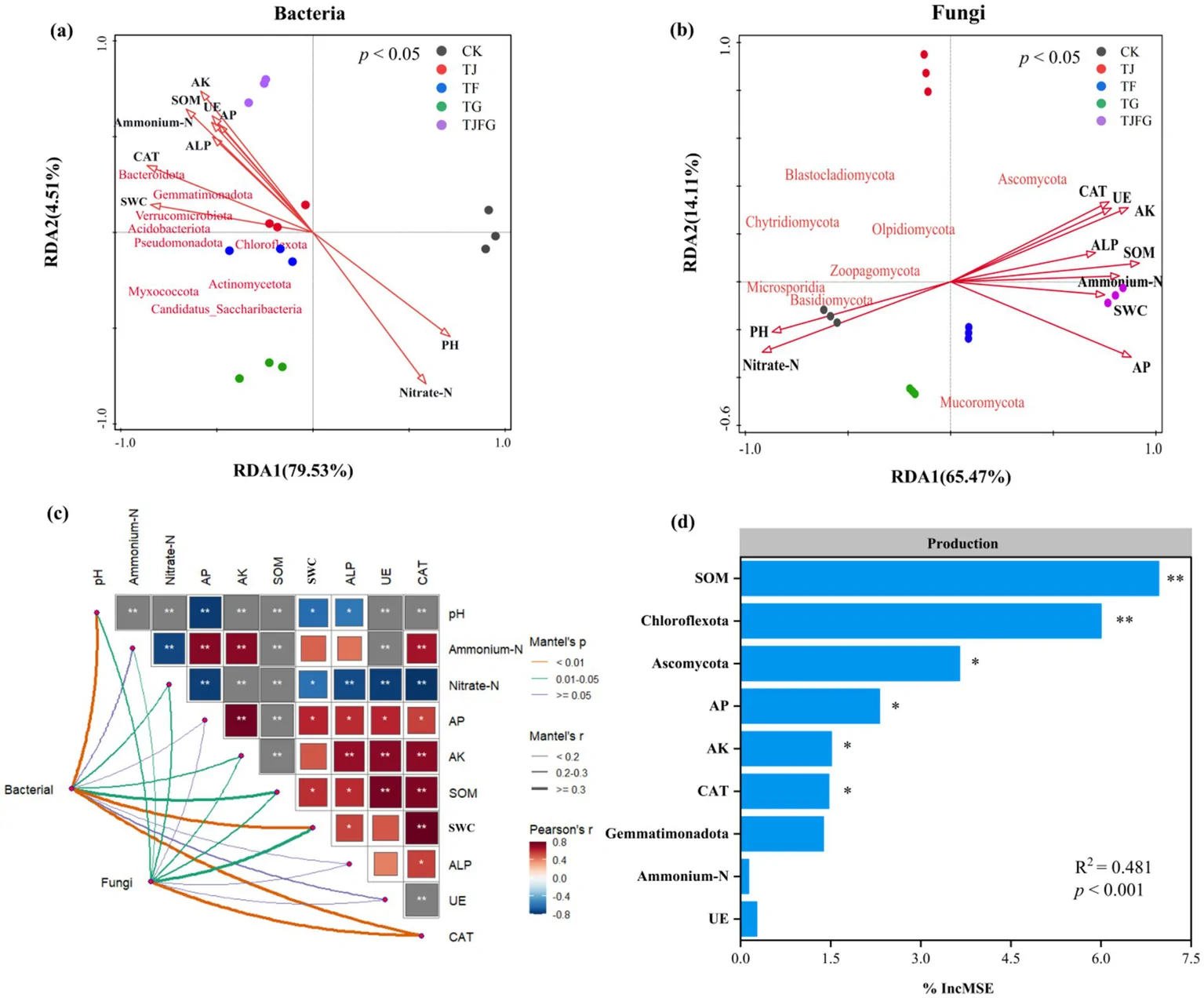
Redundancy analysis of environmental factors and rhizosphere soil bacterial (TOP 9) **(a)** and fungal (TOP 5) **(b)** community structure. Correlation analysis of soil microbial community structure and soil physicochemical properties **(c)**. Relative importance of soil microbial abundance, diversity, and physicochemical properties **(d)**. To simplify the chart, based on the results of random forest analysis, the top nine variables that contribute the most to oat yield are listed. ^*^p < 0.05; ^**^p < 0.01.

## 4 Discussion

### 4.1 Composite modifier reconstructed rhizosphere soil microbial community structure by soil nutrient regulation

As this study hypothesized, the combined use of microbial agent, organic fertilizer, and silica powder boosted the diversity of bacterial and fungal communities compared with CK (Table 2). The enhancement of diversity is closely related to the rhizosphere soil nutrients. Composite modifier triggers increases in the content of available nitrogen, AP, AK, and silicon in the soil by stimulating organic matter degradation, silicon activation, and nutrient release. The enrichment of rhizosphere nutrients optimizes the microbial habitats, driving the succession of microbial community structure and diversity (Liu et al., 2020; Zhang et al., 2016). Jin et al. (2022) found that the application of organic–inorganic compound fertilizer led to significantly higher abundance and diversity of soil bacterial communities and lower species richness and diversity of fungal communities compared with chemical fertilizer application. This is mainly because their experiment was conducted in non-salinized farmland, where the addition of organic fertilizer drove a few competitive fungi to proliferate massively, suppressing the growth of other fungi and thus lowering fungal diversity. However, the soil in this study is moderately salinized. The high pH and salinity put strong stress on native fungi. The organic modifier can supply carbon sources while alleviating salt stress, allowing more fungal groups to establish. This is why there is an increase in fungal diversity. In this study, soil pH, SWC, NO_3_^−^-N, AK, SOM, and CAT activity were all closely related to the bacterial and fungal community structure.

**Table 2:** α-diversity (Chao1, Shannon, Simpson, and ACE indexes) of microbial communities in oat rhizospheric soil under different soil modifier treatments.

Microbial group	Variety	Treatment	Chao1	Shannon	Simpson	ACE
Bacteria	Tianyan 60	CK	29,412.60 ± 1,209.31b	6.01 ± 0.29b	0.97 ± 0.06b	28,926.93 ± 1,486.71b
		TJ	30,105.88 ± 1,422.31ab	6.01 ± 0.38b	0.98 ± 0.03b	29,661.02 ± 1,228.75ab
		TF	30,226.84 ± 1,002.04ab	6.22 ± 0.09a	0.99 ± 0.04b	29,763.07 ± 921.79ab
		TG	30,063.72 ± 1,689.24ab	6.15 ± 0.31ab	1.00 ± 0.07ab	29,640.78 ± 1,689.89ab
		TJFG	30,453.94 ± 1,204.23a	6.19 ± 0.34ab	1.02 ± 0.10a	29,820.96 ± 735.67a
	Musite 2	CK	30,844.78 ± 1,270.71b	6.15 ± 0.47b	0.98 ± 0.05a	30,386.24 ± 1,219.43a
		TJ	30,968.61 ± 1,024.51ab	6.24 ± 0.38ab	1.00 ± 0.06a	30,503.75 ± 778.48a
		TF	30,813.90 ± 1,224.21b	6.13 ± 0.24b	0.99 ± 0.08a	30,388.98 ± 1,531.56a
		TG	31,007.63 ± 1,598.06ab	6.26 ± 0.41ab	0.99 ± 0.03a	30,470.53 ± 1,594.56a
		TJFG	31,361.51 ± 992.29a	6.42 ± 0.14a	1.06 ± 0.09a	30,665.74 ± 1,206.93a
Fungi	Tianyan 60	CK	715.89 ± 57.27c	4.77 ± 0.23a	0.91 ± 0.04c	673.17 ± 35.47c
		TJ	828.15 ± 75.80b	4.21 ± 0.41b	0.99 ± 0.13ab	863.67 ± 56.75b
		TF	897.70 ± 45.12ab	4.14 ± 0.26b	0.92 ± 0.09c	850.32 ± 33.29b
		TG	932.25 ± 50.23a	4.04 ± 0.15b	0.93 ± 0.08bc	932.90 ± 51.22a
		TJFG	968.67 ± 11.93a	4.11 ± 0.44b	1.01 ± 0.13a	975.30 ± 29.74a
	Musite 2	CK	813.30 ± 29.80c	4.77 ± 0.14a	0.92 ± 0.09b	802.07 ± 41.87d
		TJ	980.73 ± 16.67a	4.20 ± 0.26b	0.95 ± 0.05ab	966.07 ± 40.92b
		TF	924.50 ± 24.82b	4.68 ± 0.36a	0.96 ± 0.03ab	892.87 ± 16.97c
		TG	935.07 ± 26.44b	4.51 ± 0.18ab	0.95 ± 0.06ab	917.44 ± 46.08c
		TJFG	998.27 ± 23.85a	4.60 ± 0.45a	1.01 ± 0.05a	1,001.08 ± 28.35a

Values are expressed as mean ± standard error (*n* = 3). Different lowercase letters indicate significant differences between treatments at *p* < 0.05.

This study’s results showed that the relative abundance of dominant soil bacteria, especially Bacteroidota, increased after modifier applications. This may be because Bacteroidota is an R-strategist (Guo et al., 2020; Wu et al., 2023). Soil organic matter and nitrogen temporarily increased after modifier applications. Bacteroidota could win the competition with other microbes by virtue of its efficient nutrient absorption and metabolism system (Jia et al., 2022), quickly becoming the dominant bacteria. This aligns with the expectation in this study, i.e., properly applying soil modifiers can optimize the structure of the rhizosphere microbial community. Furthermore, the relative abundance of Ascomycota increased after modifier applications, while the relative abundance of Mucoromycota decreased. This may be because Ascomycota is a key fungal phylum indicating eutrophication, and its abundance increases with increasing soil nutrient content. Mucoromycota, on the other hand, rapidly colonize when resources are abundant, utilizing simple carbohydrates. In the early stage of modifier applications, Mucoromycota may experience a brief period of prosperity. However, as the decomposition of modifiers proceeds, simple carbohydrates are depleted, and the environment enters a stage dominated by complex organic matter. Ascomycota, due to the physical structure of mycelium and a powerful enzyme system, may have an advantage over Mucoromycota in competition (Fierer et al., 2007). Overall, these changes in community composition confirm that the combined use of microbial agent, organic fertilizer, and silicon powder optimizes the rhizosphere microbial community structure by promoting the growth of beneficial bacterial phyla like Bacteroidota and Ascomycota.

### 4.2 Collaborative regulation of rhizosphere soil properties and enzyme activity by composite modifier

In this study, rhizosphere soil properties such as pH and the contents of NH_4_^+^-N, NO_3_^−^-N, AP, AK, SOM, and SWC of the TJFG treatment were significantly higher than those of CK (Table 1). Studies have shown that microbial agents and silicon powder each have the potential to restore soil functions and enhance fertility and rhizosphere environment (Bai et al., 2020; Kearl et al., 2019; Wang et al., 2023; Zhu et al., 2022). Moreover, using them together can improve soil properties and increase fertilizer use efficiency compared with applying them individually (Zhang et al., 2023; Zhou et al., 2022). These improvements can be attributed to several factors. Firstly, the microbial agent accelerates the decomposition of organic fertilizer and promotes the reproduction of acid-producing microorganisms. Secondly, silica powder is slowly hydrolyzed in soil to form silicic acid (H_4_SiO_4_), neutralizing alkali metal ions and reducing soil pH (Houben and Sonnet, 2015). Decreased soil pH boosts the accumulation of available phosphorus and potassium by reducing phosphorus and potassium fixation and promoting mineral dissolution (Lashari et al., 2013). In addition, under low-pH environments, NH_4_^+^ is more susceptible to soil colloid adsorption, reducing volatility and leaching. The high adsorption capacity and sustained release of silica powder reduce the leaching loss and significantly improve the effectiveness of the bacterial agent and organic fertilizer (Table 1). The increase in SWC is mainly because the humic acid in organic fertilizer and silica acid produced by hydrolysis of silica powder are combined through coordination bonds to form organic–inorganic composite colloids with porous structures. Organic–inorganic composite colloids can enhance soil cohesion, humus content, porosity, and water-holding capacity (Song et al., 2020). In addition, soil enzymes are key biocatalysts for nutrient conversion and energy flow. In this study, the activity of soil ALP and UE of the TJFG treatment was significantly higher than that of CK and other modifier treatments (Figure 3). The activities of soil ALP and UE of TY 60 of the TJFG treatment were 57.22 and 54.45% higher than those of CK, respectively. The activities of soil ALP and UE of MST 2 of the TJFG treatment were 35.66 and 74.91% higher than those of CK, respectively. This may be because the increase in SOM content induced by TJFG provides more organic carbon for soil microorganisms (He et al., 2020). This shows that the composite modifier can improve soil physicochemical properties through acidification and nutrient activation. Simultaneously, it boosts the activity of multiple key soil enzymes, thus creating a favorable rhizosphere environment for plant growth.

The oat variety MST 2 showed higher NH_4_^+^-N accumulation and lower NO_3_^−^-N leaching than TY 60. Its rhizosphere soil CAT activity was also significantly higher. Based on previous studies on salt-tolerant crop genotypes (Wang et al., 2015; Zhang et al., 2005), it is speculated that this might be related to MST 2 having a more developed root system. On one hand, a well-developed root system can absorb nitrate nitrogen (NO_3_^−^-N) from deeper soil layers efficiently, reducing NO_3_^−^-N movement to the leaching layer and thus lowering the risk of nitrogen loss (Coskun et al., 2017). On the other hand, the developed roots and pore structure tend to create an oxidized environment, which promotes the growth of aerobic microorganisms. Bacteria from the phylum Pseudomonadota, which are important producers of CAT in soil (Li et al., 2023), increase in relative abundance, leading directly to higher CAT activity (Ling et al., 2022; Figure 5a).

### 4.3 Variety and modifier interactions improved oat physiology, metabolism, and quality formation through rhizosphere microecology regulation

The high pH of salinized soils can disrupt the ionic balance of crop cells and interfere with nutrient absorption and metabolic processes, ultimately adversely impacting crop yield and quality (Isayenkov and Maathuis, 2019; Munns and Tester, 2008). In this study, the application of different modifiers had significantly positive effects on the yield of TY 60. For the yield of variety MST 2, only the TF and TJFG applications showed significantly positive effects (Figure 1). The response differences between varieties mainly come from MST 2 relying more on organic matter and microbial activity in rhizosphere soil. The application of organic fertilizer enhances microbial functions and soil fertility, creating a favorable root environment for MST 2, which in turn improves its nutrient uptake efficiency. In this study, the CP and RFV of MST 2 reached their highest levels under the TJFG treatment, which were 35.10 and 5.96% higher than those of CK. This result confirms this study’s hypothesis that the composite modifier can enhance both crop yield and quality compared to single modifiers. Microbial agent and organic fertilizer provide carbon and energy for the activation of silica powder and the growth of beneficial microbial communities in rhizosphere soil. The addition of silica powder improves the rhizosphere environment and microbial activity, promoting plant root growth (Mahmood et al., 2016). Their interaction enhances the bioavailability and absorption of nitrogen and silicon in the rhizosphere soil, promotes nitrogen assimilation and amino acid conversion, thereby increasing the CP content and quality of oats (Li and Nishino, 2011). In addition, there were significant differences in oat yield, RFV, NDF, and ADF between the two varieties. The consistent response of the two varieties to modifiers across different years is mainly due to the lasting effects of soil improvement practices. After applying organic fertilizer, available nutrients can continue to be slowly released the following year through microbially mediated mineralization (Richardson et al., 2009). Microbial agents can promote the formation and stability of soil aggregates. Together with silicate powder, they can improve soil pore structure and nutrient retention capacity (Liu et al., 2024). This steadily boosts oat yield and quality in the second year and allows the genetic differences among oat varieties to be fully expressed in the improved growing conditions. Compared with TY 60, MST 2 exhibits greater growth potential and deserves wide application. Therefore, the genotypic difference in yield response to modifiers is mainly determined by the rhizosphere nutrient utilization strategy of each variety.

### 4.4 Composite modifier regulated microbial diversity to enhance soil enzyme activity and function

In this study, the application of Bacillus, organic fertilizer, and silica powder breaks the eco-balance of the microbial community and stimulates competition among indigenous microorganisms. This ultimately changes the structure of the soil microbial community and increases the Chao1 and ACE indices of the soil microbial community. In addition, the rhizosphere microbiota is the core of plant-environment interaction, driving plants’ nutrient acquisition and regulating the rhizospheric microenvironment (Trivedi et al., 2020). Microorganisms secrete various hydrolytic enzymes and oxidoreductases into the extracellular environment to decompose organic matter and obtain nutrients (Shi et al., 2019). In this study, the activities of ALP, UE, and CAT were negatively correlated with soil pH and NO_3_^−^-N content, but positively correlated with soil available nutrients (AP, AK, SOM) and SWC. Related studies have obtained consistent results (Figure 5c). This aligns with the findings of Kuzmanovic et al. (2024), i.e., a positive correlation between phosphatase activity and soil nutrient content. This suggests that boosting enzyme activity is key to enhancing soil function and productivity. Adding complex modifiers first reshapes the rhizosphere microbial community, and the increase in core functional microorganisms directly drives higher soil enzyme activity, which in turn speeds up nutrient conversion and release in the soil. Sufficient available nutrients ultimately support the oat yield and quality formation (Yang et al., 2020). The results of random forest analysis further verified this point. The abundance of Chloroflexota and Ascomycota, the contents of SOM, AP, and AK, as well as the activity of CAT, had important effects on oat yields. This indicates that improvements in soil nutrient availability and oat growth are driven by enzyme activity regulated by key microorganisms such as Chloroflexota and Ascomycota.

### 4.5 Limitations and prospects

This two-year field study was conducted at a single site. Therefore, the transferability of the conclusions to other salinized lands still needs additional validation. The small sample size (three replicates per treatment) limits the statistical power for high-throughput metagenomic sequencing. Additionally, this study did not include pairwise combinations (i.e., microbial agents + organic fertilizer, microbial agents + silicon powder, organic fertilizer + silicon powder), which made it difficult to distinguish binary interaction effects and to fully confirm the synergistic benefits among the three components. Moreover, the shotgun metagenomic sequencing used in this study relies on assembly and gene set annotation and is affected by sequencing depth and assembly quality. Thus, there are methodological limitations in analyzing low-abundance microbial groups.

It’s worth noting that while the hydrolysis of silica powder produces silicic acid, long-term continuous silica powder application also carries potential environmental risks, such as soil silicon accumulation and antagonistic interactions with trace elements like iron and molybdenum. Due to the limited period in this study, noticeable silicon accumulation and trace element imbalances were not observed. However, these risks still need to be monitored in field applications.

Furthermore, this study did not measure root morphological and physiological indicators. Therefore, the mechanisms of different oat genotypes in response to the composite modifier still need further investigation using indicators like root length density, root surface area, and root activity.

In the future, multi-site long-term trials will be conducted, with the addition of two-component combinations, alongside root morphological and physiological measurements. This will further reveal the regulatory mechanisms of the composite modifier in salinized soil–oat systems, supporting forage-based soil improvement practices in salinized lands.

## 5 Conclusion

The combined application of microbial agent, organic fertilizer, and silica powder alleviated salt stress and enhanced soil quality, oat yield, and quality. Its main mechanism of action is to regulate the diversity of rhizosphere microbial communities to increase soil enzyme activity and promote nutrient release, thereby enhancing oat yield and quality. Chloroflexota and Ascomycota had a significant impact on oat yield. In terms of soil factors, organic matter contributed the most to oat yield, followed by available phosphorus, available potassium, and catalase activity. In addition, under the salinized conditions of this trial, the oat variety MST 2 showed better growth performance and application potential than TY 60. However, its regional adaptability still needs to be confirmed through multi-site, multi-year field trials. To achieve soil health and forage yield improvement in the salinized lands of northwest China, it is necessary to select proper oat varieties and modifiers based on regional environmental conditions, balancing economic feasibility and effectiveness.

## Data Availability

The data analyzed in this study are available in the NGDC GSA (https://ngdc.cncb.ac.cn/gsa) under accession number CRA033617.
