# Supplementary material for: The combination of microbial agent, organic fertilizer, and silica powder enhances oat yield and quality through regulating rhizosphere processes
Source: Front Microbiol. 2026 Oct 15;17:1901736. doi: 10.3389/fmicb.2026.1901736 (PMC13717616; doi:10.3389/fmicb.2026.1901736)
Supplement: Supplementary file 1 [file Supplementary_file_1.docx]

**Supplementary material**

**Table S1.** Key salinization indicators of soil in the study area

| Soil layer  (cm) | Total salt content  (g kg⁻¹) | pH  (soil: water = 1:2.5) | Exchangeable sodium percentage (ESP, %) | Major soluble salt ions |
| --- | --- | --- | --- | --- |
| 0-20cm | 3.85 | 8.39 | 12.45 | Na⁺, Cl⁻, SO₄²⁻, Ca²⁺ |
| 20-40cm | 3.16 | 8.44 | 11.55 | Na⁺, Cl⁻, SO₄²⁻, Ca² |

Note: The soil data comes from measurements of mixed samples taken from the test area before the experiment, reflecting the overall spatial variation in salinity.

**Figure S1.** Monthly average values of air temperature and precipitation at the experimental site during 2023 and 2024**.**

**Table S2.** Two-way ANOVA results (*F* value and *p* value) of physicochemical properties in response to Oat variety (V) and modifier treatments (T). *, *p* < 0.05; **, *p* < 0.01; ***, *p* < 0.001.

| **Two–way ANOVA** | **pH** | |  | **NH_4_^+^-N** |  |  | **NO_3_^-^-N** |  |  | **AP** | |  | **AK** | |  | **SOM** | |  | **SWC** | |
| --- | --- | --- | --- | --- | --- | --- | --- | --- | --- | --- | --- | --- | --- | --- | --- | --- | --- | --- | --- | --- |
|  | **F** | ***P*** |  | **F** | ***P*** |  | **F** | ***P*** |  | **F** | ***P*** |  | **F** | ***P*** |  | **F** | ***P*** |  | **F** | ***P*** |
| **V** | 1.006 | 0.329 |  | 937.658*** | 0.000 |  | 48.332*** | 0.000 |  | 0.278 | 0.604 |  | 14.932** | 0.001 |  | 23.651*** | 0.000 |  | 6.514* | 0.020 |
| **T** | 8.093** | 0.001 |  | 0.047 | 0.995 |  | 0.797 | 0.545 |  | 17.613*** | 0.000 |  | 1.011* | 0.433 |  | 0.865 | 0.507 |  | 4.827* | 0.011 |
| **V×T** | 0.195 | 0.702 |  | 0.018 | 0.906 |  | 0.068 | 0.819 |  | 27.788* | 0.034 |  | 0.369 | 0.606 |  | 0.187 | 0.707 |  | 0.097 | 0.785 |

Notes: NH_4_^+^-N, soil ammonium nitrogen; NO_3_^—^N, soil nitrate nitrogen; AP, soil available phosphorus; AK, soil available potassium; SOM, soil organic matter; SWC, soil water content.

**Table S3.** Two-way ANOVA results (*F* value and *p* value) of bacterial Chao1, Shannon, Simpson, and ACE indexes in response to Oat variety (V) and modifier treatments (T). *, *p* < 0.05; **, *p* < 0.01; ***, *p* < 0.001.

| **Two–way ANOVA** | **Chao1** | |  | **Shannon** | |  | **Simpson** | |  | **ACE** | |
| --- | --- | --- | --- | --- | --- | --- | --- | --- | --- | --- | --- |
|  | **F** | **P** |  | **F** | **P** |  | **F** | **P** |  | **F** | **P** |
| **V** | 22.562** | 0.001 |  | 3.319 | 0.106 |  | 0.441 | 0.525 |  | 29.269** | 0.001 |
| **T** | 0.313 | 0.858 |  | 0.979 | 0.494 |  | 5.802* | 0.040 |  | 0.205 | 0.925 |
| **V×T** | 268.232** | 0.004 |  | 3.818 | 0.910 |  | 38.847* | 0.025 |  | 689.760** | 0.001 |

**Table S4.** Two-way ANOVA results (*F* value and *p* value) of fungal Chao1, Shannon, Simpson, and ACE indexes in response to Oat variety (V) and modifier treatments (T). *, *p* < 0.05; **, *p* < 0.01; ***, *p* < 0.001.

| **Two–way ANOVA** | **Chao1** | |  | **Shannon** | |  | **Simpson** | |  | **ACE** | |
| --- | --- | --- | --- | --- | --- | --- | --- | --- | --- | --- | --- |
|  | **F** | **P** |  | **F** | **P** |  | **F** | **P** |  | **F** | **P** |
| **V** | 1.259 | 0.294 |  | 3.265 | 0.108 |  | 0.069 | 0.799 |  | 0.840 | 0.386 |
| **T** | 3.866 | 0.085 |  | 1.287 | 0.386 |  | 7.151* | 0.027 |  | 5.874* | 0.039 |
| **V×T** | 259.803** | 0.004 |  | 1459.639** | 0.001 |  | 42.436* | 0.023 |  | 277.513** | 0.004 |
